# Symptom-based vs asymptomatic testing for controlling SARS-CoV-2 transmission in low- and middle-income countries: A modelling analysis

**DOI:** 10.1016/j.epidem.2022.100631

**Published:** 2022-12

**Authors:** Yeonsoo Baik, Lucia Cilloni, Emily Kendall, David Dowdy, Nimalan Arinaminpathy

**Affiliations:** a Department of Epidemiology, Johns Hopkins Bloomberg School of Public Health, Baltimore, MD, USA; b MRC Centre for Global Infectious Disease Analysis, School of Public Health, Imperial College London, United Kingdom; c Center for Tuberculosis Research, Division of Infectious Diseases, Johns Hopkins University School of Medicine, Baltimore, MD, USA

**Keywords:** SARS-CoV-2, Modelling, Testing

## Abstract

**Background:**

Diagnostic testing plays a critical role in the global COVID-19 response. Polymerase chain reaction (PCR) tests are highly accurate, but in resource-limited settings, limited capacity has led to testing delays; whereas lateral flow assays (LFAs) offer opportunities for rapid and affordable testing. We examined the potential epidemiological impact of different strategies for LFA deployment.

**Methods:**

We developed a deterministic compartmental model of SARS-CoV-2 transmission, parameterised to resemble a large Indian city. We assumed that PCR would be used to test symptomatic individuals presenting to outpatient settings for care. We examined how the second epidemic wave in India could have been mitigated by LFA deployment in its early stages by comparing two strategies: (i) community-based screening, using LFAs to test a proportion of the population, irrespective of symptoms (in addition to symptom-driven PCR), and (ii) symptom-driven outpatient testing, using LFAs to replace PCR.

**Results:**

Model projections suggest that a stock of 25 million LFAs, used over a 600-day period in a city of 20 million people, would reduce the cumulative symptomatic incidence of COVID-19 by 0.44% if used for community-based screening, and by 13% if used to test symptomatic outpatients, relative to a no-LFA, PCR-only scenario. Sensitivity analysis suggests that outpatient testing would be more efficient in reducing transmission than community-based screening, when at least 5% of people with symptomatic COVID-19 seek care, and at least 10% of SARS-CoV-2 infections develop symptoms. Under both strategies, however, 2% of the population would be unnecessarily isolated.

**Interpretation:**

In this emblematic setting, LFAs would reduce transmission most efficiently when used to test symptomatic individuals in outpatient settings. To avoid large numbers of unnecessary isolations, mass testing with LFAs should be considered as a screening tool, with follow-up confirmation. Future work should address strategies for targeted community-based LFA testing, such as contact tracing.

## Highlights

• Rapid and affordable testing can be critical in the response to the SARS-CoV-2 pandemic in resource-limited settings.
• We assess whether a rapid diagnostic tool would be more efficient in outpatients settings or in the general community.
• Using LFAs in outpatient settings has a greater impact than using them in the general community because it detects symptomatic cases and reduces diagnostic delays.
• In both outpatient and community strategies, using confirmatory testing, such as PCR, could avoid unnecessary isolations.

## 1 Introduction

Diagnostic testing remains a critical component in the global response to COVID-19 (Botti-Lodovico et al., 2021, Rosenthal, 2020). In addition to guiding clinical decisions, testing may also have an important impact in the community (Mina et al., 2020), offering opportunities to limit transmission through the timely identification and isolation of infectious cases (García-Basteiro et al., 2018, Blanco et al., 2016, Park et al., 2020). There remain important complexities in identifying rational strategies for limiting transmission through testing, particularly in low- and middle-income settings, where it is critical to deploy scarce resources in the most impactful way possible.

Molecular diagnostics such as reverse-transcription polymerase chain reaction (RT-PCR) tests offer highly accurate diagnosis, with over 99% sensitivity and 99% specificity (Böger et al., 2021). However, their widespread deployment has posed substantial challenges in practice: they are costly, require trained personnel to operate, and do not lend themselves to point-of-care testing, with healthcare providers often having to rely on a limited number of machines in central laboratories (Peeling et al., 2021). Reliance on RT-PCR has typically resulted in long turnaround times, and it remains infeasible to use these tests as a tool for widespread screening in the community.

The emergence of new, rapid diagnostic tests in recent months may offer important opportunities to address these challenges. Based on detection of viral antigen through lateral flow assays (LFAs), these tests can be used at the point of care with minimal training, without reliance on any laboratory infrastructure, and with fast turnaround times of less than an hour (Peeling et al., 2021). Despite reduced sensitivity and specificity compared to RT-PCR (Mina et al., 2020, García-Basteiro et al., 2018, Crozier et al.,), LFAs could – by virtue of their ease-of-use and potential widespread availability – contribute to reducing transmission through early detection and isolation of individuals with SARS-CoV-2 infection. One possible application of LFAs is to replace PCR testing, for symptomatic individuals in outpatient settings. Despite reduced sensitivity and specificity compared to RT-PCR (Mina et al., 2020, García-Basteiro et al., 2018, Crozier et al.,), LFAs could have valuable benefits by facilitating early diagnosis and isolation of individuals with SARS-CoV-2 infection, amongst those who present for care with symptoms. Alternatively, by virtue of their ease-of-use and potential widespread availability, LFAs might also be deployed at the community level, to detect individuals with asymptomatic/presymptomatic infection. This approach, already attempted in some settings in the UK (Gill and Gray, 2020) and elsewhere, could offer valuable opportunities for reducing transmission, but might come at the expense of efficiency, as fewer people are likely to test positive than if the same tests are restricted to symptomatic individuals presenting for care. In settings of fewer resource restrictions, this tradeoff may be less germane – but in many low- and middle-income countries (LMICs), an important consideration is how best to implement a limited stock of diagnostic tests. Although the price for an LFA test kit has been decreased and more kits are available for procurement now than before (UNICEF, 2021), it is important to identify which of these strategies (‘universal’ testing vs restricting to symptomatic individuals) would represent the most efficient use of LFAs in controlling transmission. We aimed to address this question using a mathematical model of SARS-CoV-2 transmission dynamics. With access to LFAs steadily expanding amongst LMICs (WHO, 2020, Team WAA, 2021), our analysis aimed to shed light on the most appropriate use of these tests for public health impact.

## 2 Methods

### 2.1 Model structure

**Fig. 1:**
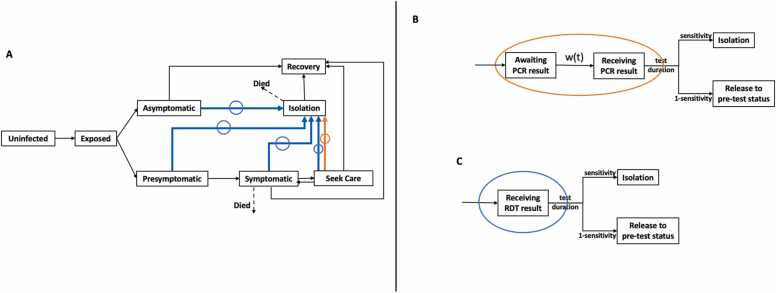
Schematic illustration of the model structure. (A) Compartments representing natural history of SARS-CoV-2, and processes involved in a test-and-isolate intervention. This structure is stratified into three age groups: children (≤ 19 years old), adults (20 – 64 years old), and older adults (≥ 65 years old). As described in the main text, we assume that asymptomatic and pre-symptomatic individuals are infectious, but potentially to a lesser extent than symptomatic cases. (In sensitivity analysis we also examine scenarios where pre-symptomatic individuals are more infectious than symptomatic individuals.) We assume that RT-PCR tests are only offered in outpatient settings, to symptomatic patients who self-present for care. LFA tests, while less sensitive, are modelled as being deployable both in outpatient settings, and amongst individuals in the community, regardless of symptoms. The arrow from ‘Seek care’ to ‘Symptomatic’ compartments show individuals who test negative, due to imperfect test sensitivity (of either RT-PCR or LFA). Arrows in red show isolation through RT-PCR testing, while arrows in blue show isolation through LFA testing, both shown in greater detail in the right-hand panels (B) Detail of RT-PCR testing. To incorporate the fact that limited PCR capacity, especially in low- and middle-income settings, often leads to delays in testing, we defined two stages for PCR testing: those who are awaiting a test, and those who have taken a test and are awaiting a result. We assume a time-dependent rate-of-transition *w(t)* from the first to the second, as described in the main text. (C) Detail of LFA testing. Unlike PCR, we assumed that there is no constraint on the number of LFA tests that can be performed per unit time, and that test results are available after a “test duration” of one hour. However, in the second part of the analysis, we assumed the availability of only a finite number of LFA tests; our analysis then examines the optimal use of this supply.

We developed a deterministic, compartmental, age-stratified model of SARS-CoV-2 transmission, illustrated schematically in Fig. 1 and described in more detail in the supporting information. Briefly, the model incorporates three different age groups: children (<19 years old), adults (19 – 64 years old) and the elderly (≥65 years old). In the main analysis, we chose demographic parameters consistent with an Indian megacity such as New Delhi, assuming an overall population size of 20 million. In sensitivity analysis, we considered a population of similar size, but with demographic parameters consistent with Kampala, Uganda, as an example of a sub-Saharan African setting. To model contacts between different age groups, we drew from synthetic contact matrices estimated by Prem et al (Prem et al., 2021).

A certain proportion of cases never develop symptoms during their infection, although they may be infectious to others; we refer to these individuals as ‘asymptomatic’. Amongst those who eventually do develop symptoms, there is evidence to suggest that they may undergo 1 – 2 days of infectiousness prior to symptom onset (Johansson et al., 2021); we refer to this as the ‘pre-symptomatic’ period. We incorporated a range of parameters for the relative infectiousness of a/pre-symptomatic infection, relative to symptomatic infection (see uncertainty, below).

The model also captures care-seeking amongst those with symptoms: we assume that a proportion of symptomatic cases will present to care in outpatient settings at some point during their symptomatic period, assuming for simplicity that this proportion remains constant through the epidemic wave We also modelled a separate, ‘non-COVID symptomatic’ (NCS) population to represent those with symptoms but without SARS-CoV-2. The purpose of modeling this population was to estimate the number of symptomatic individuals to be tested; this population does not otherwise affect compartments in Fig. 1 associated with transmission. To select the size of the NCS population, we used surveillance data from the second wave of COVID-19 in Delhi, in particular that the peak test positivity rate (TPR) was around 30%, for mainly facility-based testing (Ritchie et al., 2020) (see also supporting information for more details). To reach the same peak TPR in our simulation of the second wave required about 35,000 symptomatic individuals (without COVID-19) to seek care each day.

### 2.2 Role of testing and intervention scenarios

Consistent with conditions in Delhi by December 2020, we modelled a scenario of a city in which 30% of the population is immune, prior to the ‘second wave’ of COVID-19. We did not model vaccination: although India’s vaccination programme ultimately succeeded in covering the vast majority of the country’s population (ref), during the second wave, coverage was still low. To model the second wave we assumed a basic reproduction number $R_{0}=2.5$ (Mandal et al., 2021), incorporating any non-pharmaceutical interventions such as mask use and movement restrictions. We simulated the impact of PCR and LFA-based testing on this epidemic, as follows.

For PCR testing, we assumed that all available PCR diagnostic capacity is used only to test symptomatic patients self-presenting to outpatient settings (including both COVID and NCS populations described above). To capture the constraints arising from limited PCR capacity, we assumed that there is a limit Γon the total number of PCR tests that can be performed per day. We assumed a rate w at which samples submitted by patients actually undergo PCR testing. Borrowing concepts of carrying capacity from population ecology (Hixon, 2008), we modelled this rate in a time-dependent way as: $w\left(t\right)=w_{0}\left(1-\frac{\mathrm{PCR}\left(t\right)}{\Gamma }\right),$ where $w_{0}$ is the rate in the absence of any constraints on PCR capacity, and $\mathrm{PCR}\left(t\right)$ is the number of concurrent PCR tests being performed at time *t*. This functional form ensures that $w\left(t\right)\leq w_{0}$ throughout the simulation, while also capturing the lengthening delays that would arise when demand for PCR testing becomes too high for available capacity (Γ) to meet in a timely way. We assumed PCR sensitivity and specificity to be both 99% (Table S1).

For LFA testing, we assumed that there is no such constraint on throughput volume, as the tests themselves do not depend on laboratory capacity, and are limited only by the total number of test kits that are distributed. We assumed a finite stock of LFAs, i.e. a limit on the cumulative LFA testing that could be performed, assuming a fixed number of tests to use within a 600-day period. For simplicity we assumed LFA performance to be uniform during the natural history of disease, ignoring – for example – the relationship between LFA sensitivity and viral load (Walsh et al., 2020, He et al., 2020). We assumed LFA sensitivity and specificity to be 80% and 98%, respectively (Table S1). We examined the potential impact of different strategies for the use of this finite LFA stock, as follows.

As illustrated in Fig. 1, we modelled two different ways in which LFAs might be deployed. (i) ‘Community-based screening’, a hypothetical strategy aiming to identify as many cases as possible, including a/pre-symptomatic cases (see blue transitions in Fig. 1A and B). Under this strategy we assumed that a certain proportion of the population is tested with LFA each day, regardless of symptom status. As a baseline, we assumed no follow-up testing to confirm LFA results, whether positive or negative. We also assumed that those presenting with symptoms to outpatient settings continue to be tested with PCR. (ii) ‘Symptom-driven outpatient diagnostic testing’ (or simply ‘outpatient testing’), where LFAs are deployed in place of existing PCR to test symptomatic individuals self-presenting to outpatient settings (see orange transitions in Fig. 1A and C). Despite the lower sensitivity and specificity of LFAs compared to PCR (80% vs. 99%, and 98% vs. 99%, respectively, see Table S1 in the supporting information), an advantage of this strategy is to facilitate rapid diagnosis, without the delays relating to PCR testing (as modelled by w(t) in Fig. 1C).

As a baseline, we assumed no deployment of LFAs, and only use of PCR for symptom-driven outpatient testing. For intervention scenarios, we considered a scenario with 25 million LFA tests available to be used, over a 600-day period. This is the stock that would be needed to replace all PCR for symptom-driven outpatient testing; if used for community-based screening, this stock would be sufficient to test all individuals in the city an average of 1.25 times. We modelled the epidemiological impact under these two use cases. We also examined ‘mixed’ strategies where a given proportion p of the LFA stock is used for symptomatic patients in outpatient settings, with the remainder being used for community-based testing, at a rate sufficient to exhaust the LFA stockpile after 600 days. We examined how overall epidemiological impact (reduction in cumulative symptomatic incidence) would vary with p, thus informing whether LFAs should be used preferentially for community-based or symptomatic outpatient testing.

### 2.3 Uncertainty and sensitivity analyses

For each model parameter, we modelled uncertainty by first defining a range of plausible parameter values (Table S1). We used Latin Hypercube Sampling to draw 500 sets of parameter values from these ranges, simulating model outcomes independently on each parameter set (while controlling for the value of $R_{0}$ in the simulated second wave). From the resulting ensemble of model outcomes, we quantified uncertainty by calculating the 2.5th and 97.5th percentiles, designating the interval between these estimates as the 95% uncertainty interval (UI).

To examine the sensitivity of results for the ‘mixed’ strategy described above, we first examined how model projections would vary under different key assumptions. First, in settings where very few symptomatic individuals seek care, we would expect the impact of outpatient testing to be reduced. Accordingly, in sensitivity analysis we varied the proportion of symptomatic COVID cases that seek care for their symptoms, as well as the size of the non-COVID symptomatic (NCS) population. Second, in settings where a/pre-symptomatic individuals have a much weaker role in transmission than symptomatic cases, we would expect the impact of community-based testing to be reduced. Accordingly, we varied the infectivity of a/pre-symptomatic cases relative to symptomatic cases, as well as the proportion of infections that are asymptomatic. In addition, we relaxed our assumption in the main analysis that pre-symptomatic infection has the same infectivity as asymptomatic infection. We repeated the analysis under alternative scenarios in which pre-symptomatic infection was modeled as equally infectious, 25% more infectious, or 50% more infectious than symptomatic disease.

Finally, we explored an age-targeted scenario for community-level testing. We assumed for simplicity that children (<18 yo) are *m* times as infectious as adults, drawing values of *m* at random from the interval [0.5, 1]. We compared the impact of community-level testing targeting children, against an alternative strategy targeting adults (19 – 64 years old). We repeated this analysis using demographic parameters and a contact matrix consistent with Uganda, as an example of a sub-Saharan African setting.

## 3 Results

### 3.1 Relative impact of community vs symptom-drive outpatient testing

**Fig. 2:**
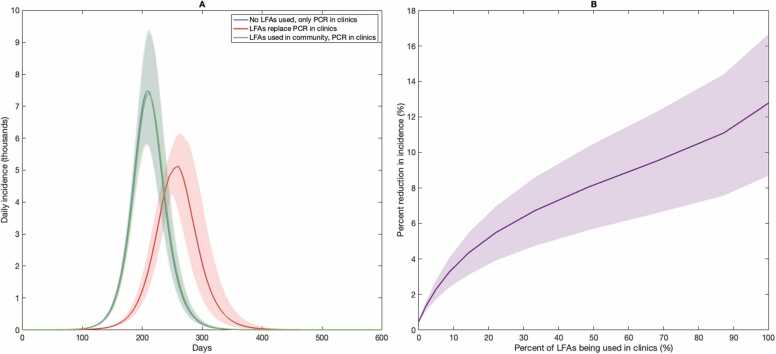
Projected epidemiological impact under different strategies for LFA deployment. (A) Illustrative examples where a fixed provision of 25 million tests is used at a steady rate over a period of 600 days to test individuals in the community, regardless of symptom status (green curve), and where the same provision of tests is used instead to replace PCR to test symptomatics self-presenting in outpatient settings (red curve). Results for cumulative symptomatic incidence are as follows: Baseline, 5.06 million (95% UI 3.82 – 6.44 million); community-level screening, 5.04 million (95% UI 3.8 – 6.42 million); symptom-drive outpatient testing, 4.4 million (95% UI 3.5 – 5.4 million). See Table 1 for additional summaries of the epidemiological impact arising from these strategies. (B) Impact of a ‘mixed’ strategy, assuming a range of scenarios for a proportion *p* of the LFA stock that is used to replace PCR for symptomatic testing in outpatient settings; we assume that any remaining LFA supplies are used for community-based screening, at such a rate as to exhaust the stock after the 600 day period.

As described above, we start with an assumed stock of 25 million LFA tests to be used over a 600-day period in a population of 20 million, equivalent to the amount that would be needed to replace all PCR testing for symptomatic patients in outpatient settings. If this stock were deployed entirely for community-based screening rather than in outpatient diagnostic settings, it would be sufficient to test 0.2% of the population per day at random, irrespective of symptoms. Such a strategy, coupled with the prompt isolation of all who test positive, could reduce cumulative symptomatic incidence in the second wave by 0.44% (95% UI 0.41 – 0.47%), compared to a scenario of no LFA testing (Fig. 2A, green curve, and Table 1).

**Table 1:** Summary of impact and unintended consequences, of the scenarios shown in Fig. 2.

	Community-based LFA testing	Symptom-driven outpatient LFA testing
Reduction in cumulative symptomatic incidence, relative to no-LFA scenario	0.44% (95% UI 0.41 – 0.47%)	12.7% (95% UI 9 – 17.2%)
Number of unnecessary isolations (as a proportion of the population) over 600-day simulation period	2.28% (95% UI 2.25 – 2.31%)	2.15% (95% UI 2.14 – 2.16%)

This estimated impact could be substantially increased if this stock of LFAs were instead used to replace PCR in symptom-driven outpatient testing, reducing cumulative symptomatic incidence 12.7% (95% UI 9 – 17.2%), relative to a scenario of no LFA testing (Fig. 2A, red curve, and Table 1). Fig. 2B shows how epidemiological impact varies as a function of the proportion of LFA tests deployed to replace PCR in outpatient settings rather than in the community. Specifically, projected epidemiological impact increases consistently with the proportion deployed in outpatient settings, suggesting that a fixed stockpile of LFAs would have a greater impact on transmission when used to replace PCR in outpatient settings, rather than to test randomly selected individuals in the community. Indeed, when all LFAs are used solely in outpatient settings, 5% of tests lead to individuals with SARS-CoV-2 being isolated. In a community testing strategy, this proportion falls to 2% (consisting of approximately 1% for symptomatic and 1% for a/-presymptomatic cases respectively), highlighting the lower efficiency of community-based testing, for identifying infection. As illustrated by the blue and green curves in Fig. S1, overall findings shown in Fig. 2 do not depend on the size of the LFA stockpile.

### 3.2 Sensitivity analyses

**Fig. 3:**
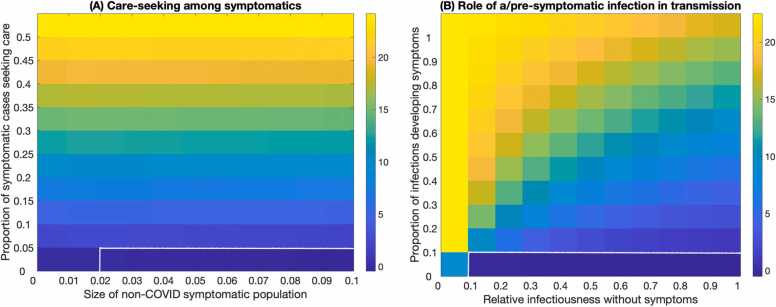
Sensitivity analysis for preferential use of LFAs in community- vs outpatient settings. In Fig. 2B, the upward-sloping curve (a positive correlation between the clinical LFA allocation and the percent reduction in symptomatic incidence) implies that LFAs would have most impact if prioritized for use in outpatient settings, whereas a downward-sloping curve would have implied that community settings should be prioritized. In the current figure, colours represent the average gradient of Fig. 2B (i.e. the gradient of a straight line connecting the left- and right-hand endpoints of the blue curve), when plotted under the different parameter combinations shown. The white line shows the isocline of zero gradient (i.e. both strategies being equally impactful). Thus lighter-shaded areas show conditions under which deployment in outpatient settings is preferred over the community, and darker-shaded areas show corresponding conditions that favor community-based testing. Overall, these results illustrate that a finite stock of LFAs would be more impactful in the community than in outpatient settings, under the following conditions: in terms of care-seeking (panel A), where the proportion of symptomatic COVID-19 cases seeking care is less than 5%, and the number of people without COVID-19 seeking care each day for respiratory symptoms is more than 3% of the population. In terms of the role of a/pre-symptomatic infection in transmission (panel B), community-based testing would be preferred where the proportion of infections developing symptoms is less than 10%; and the relative infectiousness of a/pre-symptomatic cases in the community, is at least 10% of those having symptoms.

Fig. 3 shows sensitivity analysis to examine the drivers behind these findings, specifically our assumptions regarding: the probability of developing symptoms if infected; the probability of care-seeking if symptomatic; and the transmissibility of SARS-CoV-2 during a/presymptomatic infection. In settings where fewer than 5% of symptomatic cases sought care (Fig. 3A), or where less than 10% of infections developed symptoms (Fig. 3B), community-based LFA screening was preferred. Fig. S6 in the supporting information shows examples of curves (analogous to Fig. 2B) underlying these results, showing scenarios where community-based LFA screening was preferred over outpatient testing, and vice versa. We also explored scenarios in which populations with pre-symptomatic infection were assumed to be more infectious than during the symptomatic phase, consistent with recent evidence suggesting that viral load peaks during the pre-symptomatic period (He et al., 2020). These assumptions did not qualitatively change our findings (Fig. S2), nor did calibrating the model to an age/contact structure based on a sub-Saharan African setting (Kampala, Uganda; Fig. S5). Finally, we performed sensitivity analysis under different assumptions for the maximum PCR capacity Γ. Fig. S4 illustrates that lower values for Γ are associated overall with greater impact of using LFAs to replace PCR for clinic-based testing, and vice versa. Nonetheless, the overall qualitative result shown in Fig. 2B – that LFAs would have greater impact if preferentially used to test symptomatic individuals in outpatient settings – remains unaffected by these alternative scenarios.

### 3.3 Risk of false-positive diagnoses

Table 1 also highlights the potential unintended consequences of widespread LFA testing: in particular, the prohibitive number of false-positive test results (and thus unnecessary isolations) that would occur. For example, in our community-based screening scenario, each person was screened an average of 1.25 times over the 600-day period, with assumed 98% specificity, resulting in around 2.28% of the population being asked unnecessarily to self-isolate over the 600-day period of the simulation. If instead all LFAs were used to test symptomatic outpatients, around 2.15% of the population would unnecessarily self-isolate over the 600-day period (though these individuals would be symptomatic). Arising from the imperfect specificity of LFA, these unintended consequences could be mitigated by treating LFA as a screening tool and incorporating additional tests to confirm LFA-positive results.

### 3.4 Age-specific strategies for community-level testing

Fig. S7 illustrates that the differences in the population age structure can have important implications for community-level testing strategy: in an India-like setting, it is more impactful to concentrate testing effort in adults, while in a Uganda-like setting, it is more impactful to do target children. However, the overall epidemiological impact remained comparable between these settings.

## 4 Discussion

The emergence of simple-to-use, rapid diagnostic tools has raised questions about whether they could feasibly be used to expand the detection of SARS-CoV-2 beyond those with symptoms. Our analysis shows how such strategies, unless targeted to asymptomatic individuals who have a particularly high risk of infection, are unlikely to represent the most efficient use of LFAs. First, as long as at least 10% of people with COVID-19 develop symptoms, and at least 5% of those with symptomatic COVID-19 seek testing, a given supply of LFAs would generally have a greater impact on transmission when used as a replacement for PCR in outpatient settings, to test individuals self-presenting with symptoms, rather than to screen randomly selected people in the community (Fig. 2, Fig. 3). Second, when deployed at a population level, whether in the community- or outpatient-based settings, LFAs should either achieve very high specificity when used in these settings or be used as a screening tool, with individuals who test positive requiring subsequent diagnostic confirmation (Table 1). Given recent initiatives to facilitate the procurement of large volumes of LFAs for use in resource-limited settings (UNICEF, 2021, WHO, 2020, Team WAA, 2021), our analysis provides some insights into how these tests could best be deployed to control transmission.

Our finding that using LFAs as a stand-alone test without confirmation at a population level could cause prohibitive numbers of false positive diagnoses underlines similar findings from a recent systematic review (Dinnes et al., 2021), and echoes WHO guidance for the use of LFAs (Organization WH., 2020a). While we have modelled hypothetical diagnostic tests with performance consistent with the WHO target product profiles (Organization WH., 2020b), we expect these results to remain qualitatively unchanged among the currently available tests. Indeed, a recent Cochrane review and other empirical studies highlighted that the sensitivity of these tests is lower amongst asymptomatic cases than amongst those with symptoms (Dinnes et al., 2021, Wagenhäuser et al., 2021, Jian et al., 2022, Fernandez-Montero et al., 2021, Döhla et al., 2020), suggesting that LFAs for rapid screening combined with RT-PCR as a confirmatory tool, sequential LFA testing, or our approach of outpatient-based deployment would be favored even more strongly than community-based deployment than modelled here. While our analysis has focused on a population consistent with a South-Asian city, additional analysis showed similar results for a population with age structure consistent with a city in sub-Saharan Africa (see Fig. S5). Moreover, in this analysis we have focused on urban settings. If transmission is less intense in rural settings, we would expect the impact of both community- and outpatient-based testing strategies to be higher than shown in Fig. 2. Nonetheless, because the drivers shown in Fig. 3 are not dependent on transmission intensity, we would expect our overall findings – for the relative value of these two strategies – to hold in rural settings as well. Our analysis does not address alternative, risk-focused strategies for LFA deployment: for example, areas showing high infection activity have been designated as ‘containment zones’ in New Delhi and other major cities in India (Department of Revenue, 2021), with the immediate implementation of control measures including local movement restrictions, as well as intensified testing. The use of LFAs for community-based screening may be more strongly supported in such high-infection situations than the risk-agnostic approach we have examined here. Other potential uses for LFAs include surveillance at the community level to identify hotspots or testing amongst close contacts of confirmed cases of COVID-19. Moreover, LFAs might be used in a dynamic way, adjusting their deployment in response to local epidemiological conditions (for example, depending on whether infection is stably low, or rapidly increasing). Focusing as it does on the potential value of addressing asymptomatic and presymptomatic infection, our simple model does not address these possibilities; examining these strategies is an important area for future analysis.

There are additional limitations that should be considered when interpreting our analysis. First, we have focused here on a specific use case for LFAs, that of reducing opportunities for transmission through early detection and isolation. Our analysis therefore does not speak to a range of other possible uses or benefits of testing, including to guide clinical management for patients who have or are at risk for severe disease to inform outbreak response in congregate or occupational settings. Indeed, it should be emphasized that our results for deployment in outpatient settings are focused on those with mild symptoms; the use of highly sensitive and specific molecular tests is likely to have strong justification in tertiary care settings for the testing of individuals with severe disease and risk of mortality. Previous modelling work has preliminarily addressed these use cases (Ricks et al., 2021, Larremore et al., 2021); these results could be further refined as more data becomes available on the performance of existing and emerging LFAs. Second, although we have modelled community testing as a random, untargeted approach, in practice it might be guided by strategies to improve its efficiency, for example excluding individuals with known, recent history of SARS-CoV-2 infection. More dynamic scenarios for the allocation of tests in community-based screening were beyond the scope of our study, but should be explored to provide better operational guidelines. Third, we have used certain simplifying assumptions in modelling LFA and PCR performance. For example, we assume constant test sensitivity over the course of infection, thereby not capturing variation in viral and antigen load over time (Mina et al., 2020). We have also assumed a constant care-seeking rate for the duration of the epidemic wave; if in reality symptomatic individuals are increasingly likely to seek care as an epidemic progresses, this would tend to promote the impact of outpatient testing. Fourth, our analysis does not address implementation considerations. For example, if LFAs are to be used in place of PCR for future outbreak response in outpatient settings, there remain important questions (not addressed by our study) about the supply and distribution of these tests, as well as the role of existing PCR capacity. Finally, while LFAs typically have a lower per-test cost than PCR, a full costing exercise is beyond the scope our current analysis: we therefore do not capture the potential economic benefits of using LFA in place of PCR in outpatient settings. These limitations notwithstanding, our primary findings were robust to a broad range of parameter assumptions and sensitivity analyses.

As vaccination efforts against SARS-CoV-2 scale up across the world, widespread testing will remain a critical part of the pandemic response. With currently available tests raising important trade-offs of access and speed versus accuracy, rational and systematic deployment of these tests will be key in maximizing their population benefits. Our results highlight how – under constraints of testing capacity – the impact of LFAs when used for symptomatic testing could outweigh their potential value in identifying asymptomatic/presymptomatic infection.

## CRediT authorship contribution statement

**Yeonsoo Baik**: Formal Analysis, Methodology, Software, Writing – original draft, Writing – review & editing. **Lucia Cilloni:** Formal Analysis, Methodology, Resources, Software, Visualization, Writing – original draft, Writing – review & editing. **Emily Kendall:** Conceptualization, Writing – review & editing, Supervision. **David Dowdy:** Conceptualization, Writing – review & editing, Supervision. **Nimalan Arinaminpathy:** Conceptualization, Funding acquisition, Methodology, Supervision, Writing – original draft.
