# Supplementary material for: Symptom-based vs asymptomatic testing for controlling SARS-CoV-2 transmission in low- and middle-income countries: A modelling analysis
Source: Epidemics. 2022 Dec;41:100631. doi: 10.1016/j.epidem.2022.100631 (PMC9511882; doi:10.1016/j.epidem.2022.100631)
Supplement: Supplementary file 1 — Supplementary material [file mmc1.docx]

**Diagnostic strategies to control transmission of SARS-CoV-2 in low- and middle-income countries: a modeling analysis**

**Supplementary Text 1**

Yeonsoo Baik*, Lucia Cilloni*, Emily Kendall, David Dowdy, Nimalan Arinaminpathy

* Contributed equally

Table of Contents

[1. Model specification 2](#_Toc111801215)

[1.1 Overview 2](#_Toc111801216)

[1.2 Governing equations 2](#_Toc111801217)

[1.3 Model execution 4](#_Toc111801218)

[2. Additional figures 9](#_Toc111801219)

[3. Examining age-specific strategies for community-level testing 15](#_Toc111801220)

[4. References 17](#_Toc111801221)

# **1. Model specification**

## 1.1 Overview

We constructed a deterministic, age-structured, compartmental transmission model of SARS-Cov-2. As illustrated in **Figure 1**, upon infection, individuals move into an exposed (non-infectious) state, from which they then progress to either asymptomatic or pre-symptomatic infection (both infectious states). Individuals with pre-symptomatic infection go on to develop symptoms. A certain proportion of those with symptoms seek care in primary care settings, where there may be tested for SARS-CoV-2. Upon a positive test result (depending on the sensitivity of the test), individuals are put into isolation, from which they either recover or die.

## 1.2 Governing equations

In the following equations, indices $a=1,2,3$ denote age groups <18 years, 19-64 years and >65 years, respectively. Model parameters are all defined and listed in **Table S1**, below.

*Susceptible*

$$\frac{dS_{a}}{dt}=-\lambda_{a}\left( t \right)S_{a}-h_{a}\left( 1-\sigma_{LFA} \right)S_{a}$$

*Exposed*

$$\frac{dE_{a}}{dt}=\lambda_{a}\left( t \right)S_{a}-\theta E_{a}-h_{a}\left( 1-\sigma_{LFA} \right)E_{a}$$

*Asymptomatic disease*

$$\frac{dA_{a}}{dt}=\left( 1-\kappa_{a} \right)\theta E_{a}-\gamma A_{a}-h_{a}s_{LFA}A_{a}$$

*Pre-symptomatic disease*

$$\frac{dP_{a}}{dt}=\kappa_{a}\theta E_{a}-\eta P_{a}-h_{a}s_{LFA}P_{a}$$

*Symptomatic disease*

$$\frac{dI_{a}}{dt}=\eta P_{a}-\left( \gamma+\mu_{a}+c \right)I_{a}-h_{a}s_{LFA}I_{a}$$

*Outpatient settings: awaiting access to PCR*

$$\frac{dV_{a}}{dt}=cp_{PCR}I_{a}-\left( \gamma+\mu_{a} \right)V_{a}-w_{PCR}\left( t \right)V_{a}$$

where $w_{PCR}\left( t \right)$ is a time-dependent rate of access to PCR (illustrated in **Figure 1C** in the main text), and is evaluated as:

$$w_{PCR}\left( t \right)=w_{0}\left[ 1-\frac{\left( v+\sum_{a} V_{a} \right)}{\Gamma} \right]$$

Here $w_{0}$ denotes the ‘baseline’ rate of having access to PCR (i.e, in the absence of any access constraints); $v$ denotes the number of symptomatic individuals awaiting PCR confirmation who do not have SARS-CoV-2 (see below); and $\Gamma$ denotes PCR capacity, represented as the maximum number of PCR tests that can be performed at one time.

*Outpatient settings: awaiting PCR test results (pending turnaround time)*

$$\frac{dW_{a}}{dt}=w_{PCR}V_{a}-\left( \gamma+\mu_{a} \right)W_{a}-\tau_{PCR}W_{a}$$

*Outpatient settings: awaiting LFA test results (pending turnaround time)*

$$\frac{dL_{a}}{dt}=c\left( 1-p_{PCR} \right)I_{a}-\left( \gamma+\mu_{a} \right)L_{a}-\tau_{LFA}L_{a}$$

*Isolation amongst those with SARS-CoV-2*

$$\frac{dJ_{a}}{dt}=h_{a}s_{LFA}\left( A_{a}+P_{a}+I_{a}+D_{a} \right)+\left[ \tau_{PCR}s_{PCR}W_{a}+\tau_{LFA}s_{LFA}L_{a} \right]-\rho J_{a}$$

*Recovery*

$$\frac{dR_{a}}{dt}=\gamma\left[ A_{a}+I_{a}+D_{a}+J_{a} \right]-h_{a}\left( 1-\sigma_{LFA} \right)R_{a}$$

*Force of infection*

$$\lambda_{a}\left( t \right)=\frac{\beta\sum_{b} m_{ab}\xi_{b}[{\phi A}_{b}\left( t \right)+\phi P_{b}\left( t \right)+I_{b}\left( t \right)+V_{b}\left( t \right)+W_{b}\left( t \right)+L_{b}(t)]}{N_{a}(t)}$$

where $\xi_{b}$ is the infectivity of individuals in age group $b$, relative to adults aged 19-64 years (a factor incorporated to allow for lower infectivity amongst children); $\phi$ is the infectivity of asymptomatic/presymptomatic infection relative to symptomatic infection; and $N_{a}=S_{a}+E_{a}+P_{a}+I_{a}+V_{a}+W_{a}+L_{a}+J_{a}+R_{a}$.

The contact matrix used in the model is shown below.

|  | *Child* | *Adult* | *Elder* |
| --- | --- | --- | --- |
| *Child* | 2.4 | 0.97 | 0.05 |
| *Adult* | 1.5 | 3.7 | 0.13 |
| *Elder* | 0.09 | 0.14 | 0.02 |

Contact matrix for Delhi-like setting, drawn from [1].

Non-COVID symptomatic population

As described in the main text, we also modelled a symptomatic population without COVID-19 infection, to facilitate estimation of the total tests performed in clinic-based settings (including on those not having COVID-19). Here, we use lower-case state labels to distinguish this symptomatic population from those with SARS-CoV-2.

*Outpatient settings: awaiting access to PCR*

$$\frac{du}{dt}=\epsilon p_{PCR}-w_{PCR}(t)u$$

where the ‘queuing’ rate $w_{PCR}(t)$ is as defined above.

*Outpatient settings: awaiting PCR test results (pending turnaround time)*

$$\frac{dv}{dt}=w_{PCR}\left( t \right)u-\tau_{PCR}v$$

*Outpatient settings: awaiting LFA test results (pending turnaround time)*

$$\frac{dl}{dt}=\epsilon\left( 1-p_{PCR} \right)-\tau_{LFA}l$$

*Unnecessary isolation amongst those without SARS-CoV-2*

$$\frac{dj}{dt}=h_{a}\left( 1-\sigma_{LFA} \right)\left( S_{a}+E_{a}+R_{a} \right)+\tau_{PCR}\left( 1-\sigma_{PCR} \right)v+\tau_{LFA}\left( 1-\sigma_{LFA} \right)l$$

The parameter $\epsilon$ denotes the (absolute) rate of presentation for care; its magnitude governs the prevalence of SARS-CoV-2, amongst those presenting for care. To fix this value, we drew from data in India’s second wave of COVID-19, during the spring of 2021, where test positivity rate typically peaked at around 30%, for mainly facility-based testing [2]. Accordingly, we simulated a second wave of COVID-19 (i.e. in the presence of pre-existing immunity, but absence of any interventions) using the governing equations above. We adjusted the value of $u$ in order to yield a 30% prevalence of SARS-CoV-2 at the epidemic peak among those being tested in clinic-based settings, using the relationship:

Prevalence in clinic-based settings = $\int_{\tau} c\sum I_{a}dt/(\int_{\tau} \left( c\sum I_{a}+\epsilon\right)dt,$

where $\tau$ is the one-week interval when the first wave peaks.

## 1.3 Model execution

We drew 500 parameter sets at random from the intervals shown in **Table S1**, using Latin hypercube sampling. For each such parameter set, we first determined the value of $\beta$ (per-contact transmission rate) needed to give a value of $R_{0}=2.5.$ To simulate a second wave of COVID-19, consistent with what has been occurring across many Indian cities in the spring/summer of 2021, we set initial conditions as a disease-free equilibrium where 30% of each age group is assumed to occupy the recovered compartment (consistent with seroprevalence surveys in India), and the remainder to occupy the susceptible compartment. We then initiated infection by perturbing this equilibrium, through introducing an infectious case in the population. We then simulated the epidemic forward in time.

We modelled the deployment of LFAs in community-based settings through the parameter $v_{a}$ in the equations above, which is the per-capita rate of community-level testing with LFA, assuming in the main text that testing is independent of age (although see sensitivity analysis, below).

We modelled the deployment of LFAs in clinic-based settings through the parameter $p_{RDT}$ in the equations above, which denotes the proportion of clinic visits that receive testing with PCR (we assumed the remainder to be tested by LFA).

We therefore estimated the total number of LFA tests being used, over a given time interval $\tau$, as:

$$\text{Number LFA tests}=\int_{\tau} \left( \Sigma_{a}h_{a}N_{a} \right)+\Sigma_{a}c\left( 1-p_{PCR} \right)I_{a}+\epsilon\left( 1-p_{PCR} \right)dt$$

For a given value of $p_{PCR}$, and taking $h_{a}$ to be a constant $h$across age groups, we adjusted the value of $h$to yield a given usage of LFAs over the time period $\tau$.

We repeated simulations for each of the 1500 parameter sets, quantifying uncertainty in model projections as the 2.5^th^, 50^th^ and 97.5^th^ percentiles, denoting the interval between the former and latter as the 95% uncertainty intervals.

**Table S1. List of model parameters.**

| Parameter | | Symbol | Value (95% uncertainty interval) | Reference/Notes |
| --- | --- | --- | --- | --- |
| *COVID-19 natural history* | | | | |
| Infection rate | | $\beta$ | Estimated in order to give $R_{0}$=2.5 | |
| Per-capita rate of incubation | | $\theta$ | 1/5 | Chosen to give an average incubation period of 5 days [3,4] |
| Per-capita rate of symptom development from pre-symptomatic stage | | $\eta$ | 1 | Chosen to give an average pre-symptomatic period of 1 day [4,5] |
| Per-capita rate of spontaneous recovery | | $\gamma$ | 1/5 | Chosen to give an average infectious period of 5 days [6] |
| Mortality rate | | $\mu_{a}$ | $2\times{10}^{-5}$, children  0.0006, adults  0.0137, elderly | Calculated using case fatality rate estimates  0.0001, children  0.003, adults  0.064, elderly [4] |
| Proportion of infections that develop symptoms | | $\kappa_{a}$ | (0.125 – 0.6), children  (0.5 – 0.8), adults  (0.5 – 0.8), elderly | For children, we allow a reduced probability of infections developing symptoms [4] |
| Infectivity of symptomatic disease, relative to adults | | $\xi_{a}$ | (0.50 – 1), children  1, adults  1, elderly | [7] |
| Infectivity of a/pre-symptomatic relative to symptomatic | | $\phi$ | 0.67 (0.5 – 1) | [3,8,9] |
| *Health system* | | | | |
| Rate of care-seeking amongst those with symptomatic SARS-CoV-2 | | *c* | (0.01 – 0.1) | Chosen to yield a given proportion of symptomatic individuals seeking care, as this proportion is $\frac{c}{c+\gamma+\mu_{a}}$ [10]. With $\gamma$ having an assumed value of $1/5,$ the upper limit of 0.1 on $c$ corresponds to an upper limit of roughly 1/3 of symptomatic individuals (including those with mild illness) seeking care for their symptoms. |
| Number of people with symptoms but without SARS-CoV-2, presenting to outpatient settings for care per day | | $\epsilon$ | 35,000 | Chosen so that, at the peak of the second wave, there is a 30% prevalence of SARS-CoV-2 amongst symptomatics presenting for care [2] |
| PCR testing capacity | | $\Gamma$ | 1,500 | Maximum number for daily PCR tests in a Delhi-like setting [11] |
| Baseline rate of access to PCR, amongst symptomatics in outpatient settings | | $w_{0}$ | 1/2 | Chosen to give an average delay to access testing, at baseline, of 2 days [2] |
| Turnaround time once test initiated | | $\tau_{PCR}$ | 1 | Corresponds to turnaround times of 1 hour for LFA and  1 day for PCR  [12] |
|  |  | $\tau_{LFA}$ | 24 |  |
| Per-capita rate of LFA testing via community screening | | $h_{a}$ | Dependent on scenario | We assume that testing occurs independent of age, so that $h_{a}=h$ for a scenario-specific rate $h$ |
| Proportion of clinic visits that receive PCR testing | | $p_{PCR}$ | Dependent on scenario | We assume that the remainder receive LFA testing (if $p_{PCR}\neq1$) |
| PCR test characteristics | Test sensitivity | $s_{PCR}$ | 0.99 | [2,13–15] |
|  | Test specificity | $\sigma_{PCR}$ | 0.99 | [13,14] |
| LFA test characteristics | Test sensitivity | $s_{LFA}$ | 0.80 | [16] |
|  | Test specificity | $\sigma_{LFA}$ | 0.98 | [16] |
| *Demographics* | | | | |
| Initial seroprevalence prior to second wave, Delhi-like setting | |  | 0.30 | [17] |
| Initial seroprevalence prior to second wave, Kampala-like setting | |  | 0.05 | [18] |
| Population size, Delhi-like setting | | $N$ | Children/young adults: 7,554,53  Adults: 11,954,901  Elderly: 818,671 | [19,20] |
| Population size, Kampala-like setting | | $N$ | Children/young adults: 1,896,869  Adults: 1,335,999  Elderly: 65,495 | [21,22] |

# **2. Additional figures**


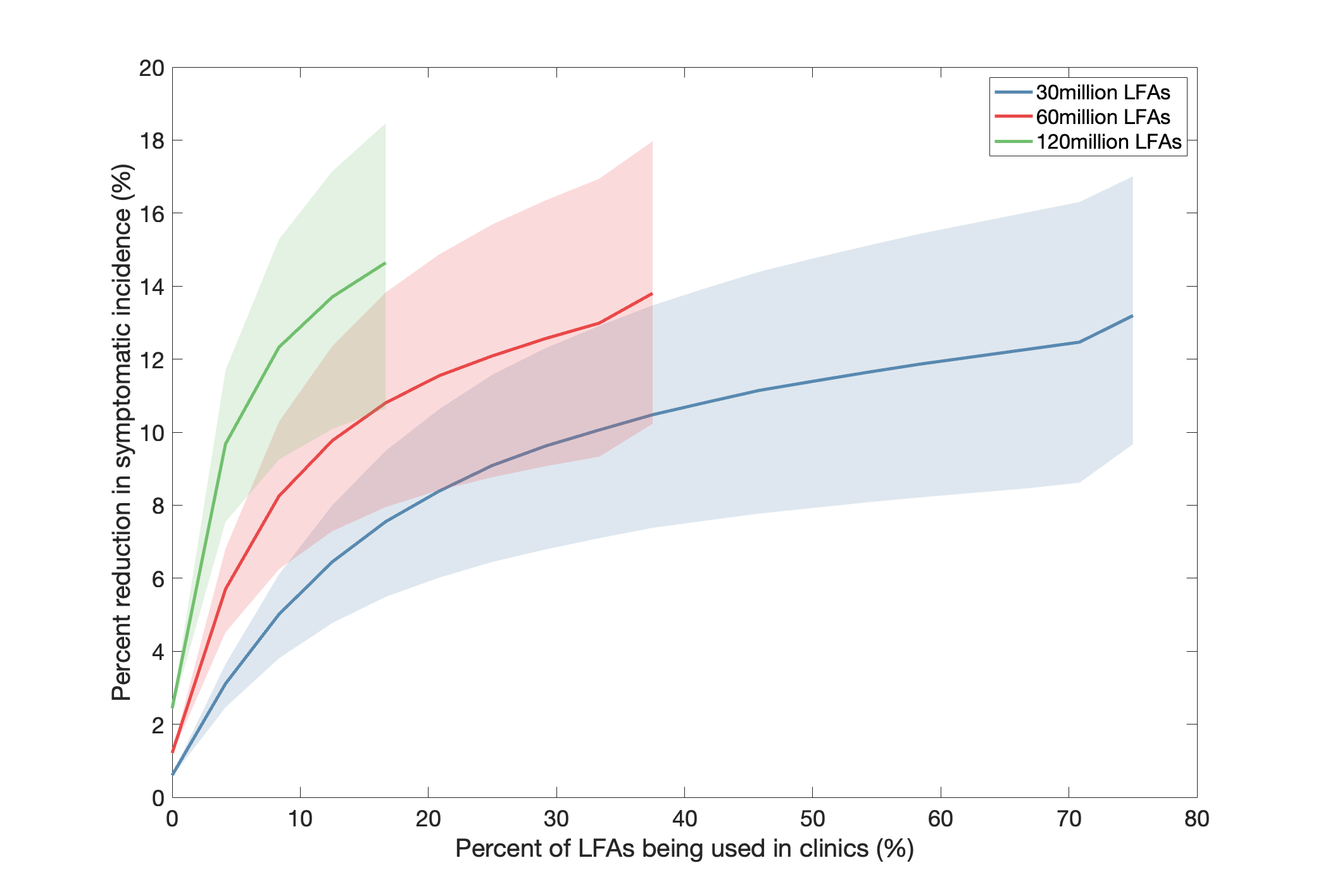


**Figure S1. Comparing strategies for prioritizing the use of LFAs in clinical vs community settings.** Whereas Figure 2 in the main text examines the case where LFAs can be used in only one of two modes (testing symptomatics self-presenting to the clinic vs individuals in the community regardless of symptoms), here we compare strategies where a given supply of LFAs, to be fully spent over a 600-day period, can be partially deployed in clinics, with the remainder being used in the community. The x-axis shows the proportion of the LFA supply being allocated to replace PCR in clinics in this way, assuming that the remainder of the supply is deployed in the community, and used at a uniform rate over 600-day period.

**Figure S2. Relative value of clinic- vs community-based deployment of LFAs, under alternative assumptions for the role of pre-symptomatic infection in transmission.** For this analysis we held the infectivity of pre-symptomatic infection at a constant level *k* relative to symptomatic infection, and then reconstructed **Figure 3B** in the main text to map the relative value of clinic- vs community-based deployment of LFAs, in relation to the role of asymptomatic infection. Areas under bold white lines show parameter regimes where community-based deployment would have greater impact than clinic-based deployment: these results illustrate that this area is not substantially altered, when allowing *k* to vary from 1 (top left panel) to 2 (bottom right).


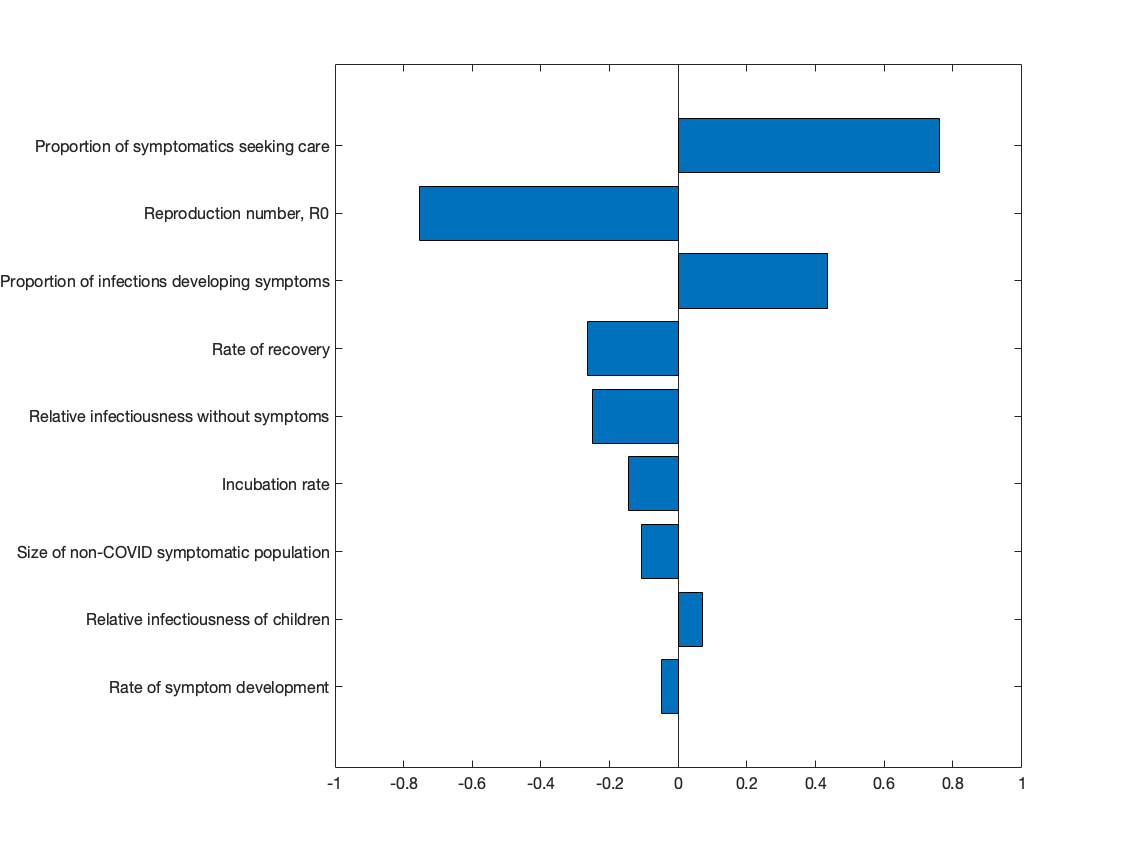


**Figure S3. Multivariate sensitivity analysis to model parameters.** We calculated the partial rank correlation coefficients (PRCC) across all model parameters with respect to the maximum achievable impact (percent cases averted) for a fixed provision of 25 million LFAs, shared equally between outpatient settings and community-level over the intervention period. Larger bars show parameters to which model projections for cases averted are most sensitive.

**
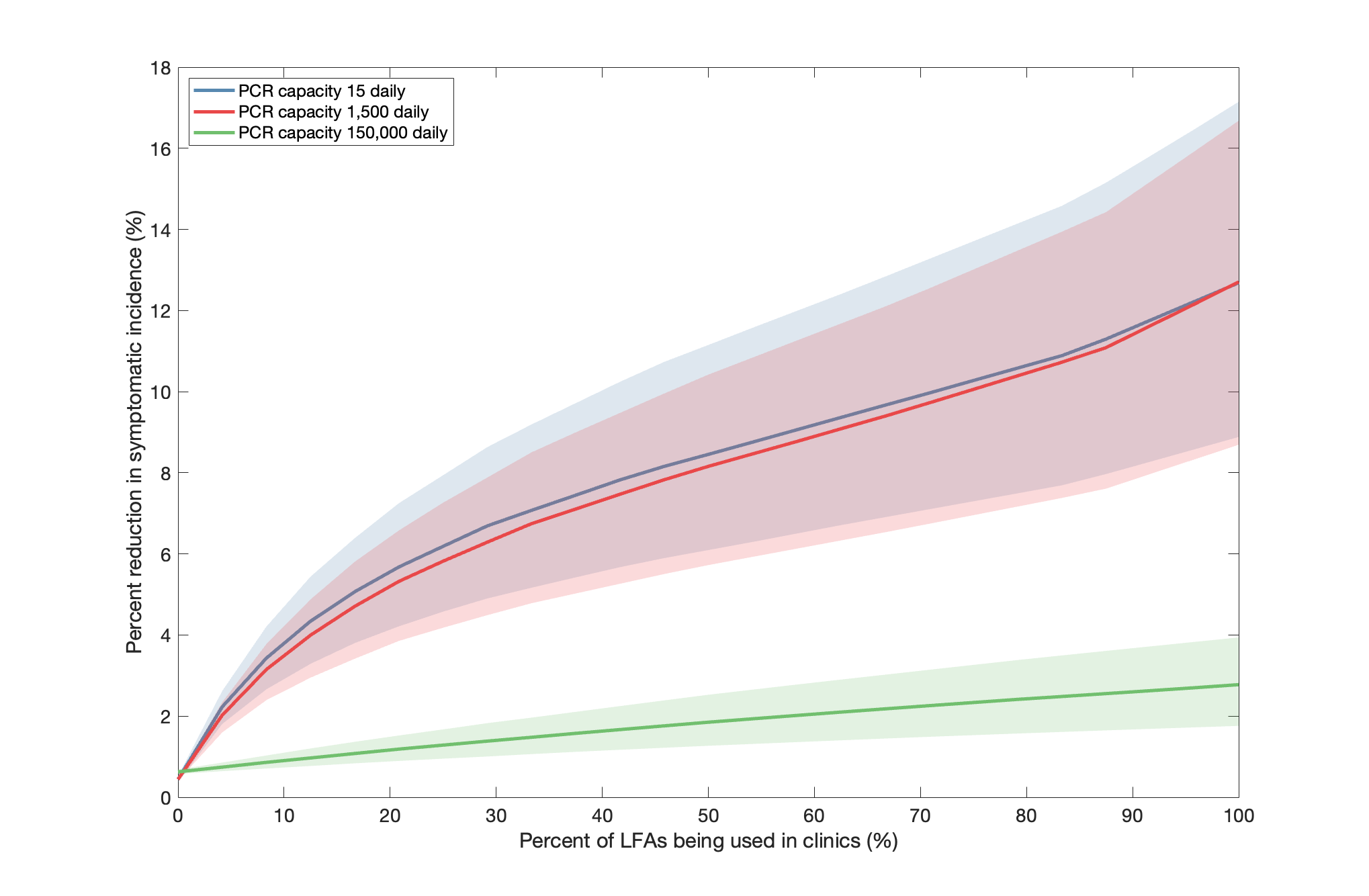
**

**Figure S4. Sensitivity analysis to assumed capacity of PCR in outpatient settings.** Figure shows same results as plotted in Figure 2B in the main text (red curve), but assuming lower and higher levels (green and blue curves, respectively) of existing PCR capacity in outpatient settings. Consistent with Fig.2B, these results show greater impact on the epidemic when LFAs are used preferentially to replace PCR to test symptomatic individuals in outpatient settings, rather than for symptom-agnostic community screening. However, the figure illustrates that this impact is generally greater in settings with higher levels of existing PCR capacity (green curve). In such settings, the incremental benefit that LFAs can provide is diminished by the ready availability of existing PCR.


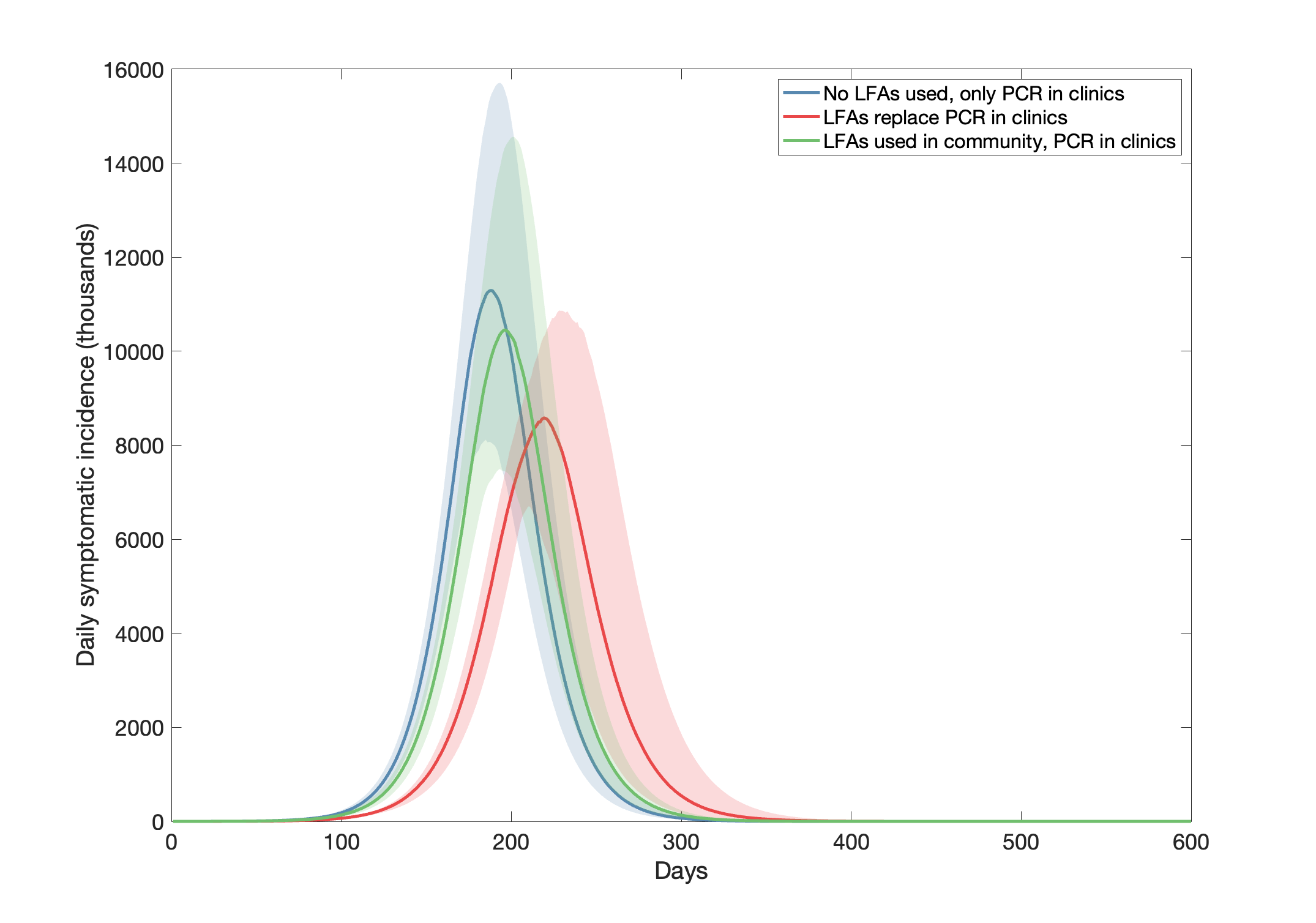


**Figure S5. Projected epidemiological impact in a Kampala-like setting**. We repeated the main analyses after re-calibrating the model to be representative of the age and contact structure of the population of Kampala, Uganda. Here, the impact of replacing PCR with LFAs in outpatient settings is of 9.5% percent symptomatic incidence reduction (95% CI 7-14%), while using all LFAs in the community and PCR in the clinics reduces symptomatic incidence by 2.7% (95% CI 2.6 - 2.8%).

**
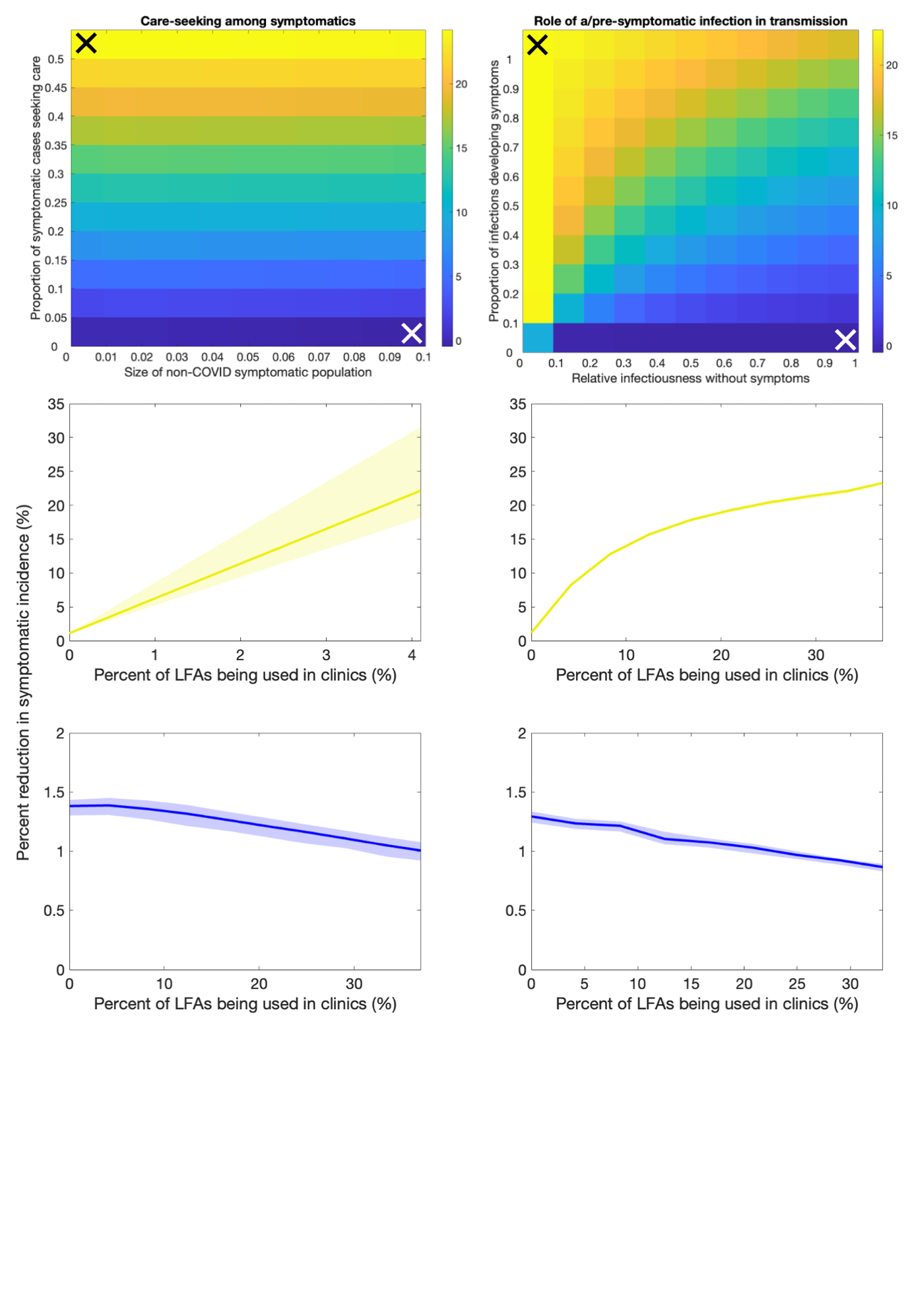
**

**Figure S6. Illustrative curves from Figure 3.** Top panels show the same plots as in Figure 3 in the main text, for straightforward reference (the left-hand figure being the same as Figure 3A, and the left-hand figure the same as Figure 3B). In these panels, the black cross shows an example parameter combination under which it is epidemiologically more impactful to prioritise LFAs in outpatient settings: the underlying curves of impact vs preferential use in outpatient settings are shown in the middle panels, illustrating a positive gradient. Likewise, the white cross shows an example parameter combination under which it is epidemiologically more impactful to prioritise LFAs for community screening: the underlying curves of impact vs preferential use in outpatient settings is shown in the bottom panels, illustrating a negative gradient.

# **3. Examining age-specific strategies for community-level testing**

In the main text we simulated testing strategies irrespective of age, while using demographic parameters consistent with a South Asian city. Although our main text results illustrate that community-level testing is less efficient at reducing transmission than clinic-based testing, it may nonetheless be possible to maximize the impact of community-level testing by targeting specific age groups, that may be most associated with transmission. As mentioned in the Methods, we assumed for simplicity that children (<18 yo) are *m* times as infectious as adults and elders, drawing values of *m* at random from the interval [0.5, 1], at the same time as sampling from the other parameter ranges in **Table S1**.

We compared the impact of community-level testing targeted at those under 18 years old, against an alternative strategy targeted at those aged 19 – 64 years old (**Figure S5**). We also repeated these projections using demographic and contact matrix parameters consistent with Uganda, as an example of a sub-Saharan African setting (shown below).

|  | *Child* | *Adult* | *Elder* |
| --- | --- | --- | --- |
| *Child* | 3.6 | 0.6 | 0.01 |
| *Adult* | 2.4 | 2.1 | 0.02 |
| *Elder* | 0.2 | 0.1 | 0.002 |

Contact matrix for Kampala-like setting.

**Figure S7. Projected impacts of age-specific strategies for LFA testing in the community.** We used demographic parameters, and age-specific contact matrices [1], consistent with **(A)** Delhi (India), as in the main text, and **(B)** Kampala (Uganda), as an example of a sub-Saharan African setting. Blue lines show the projected impact of focusing testing on children (under 18 years old), while red lines show the projected impact of focusing testing on adults (19 – 64 years old).


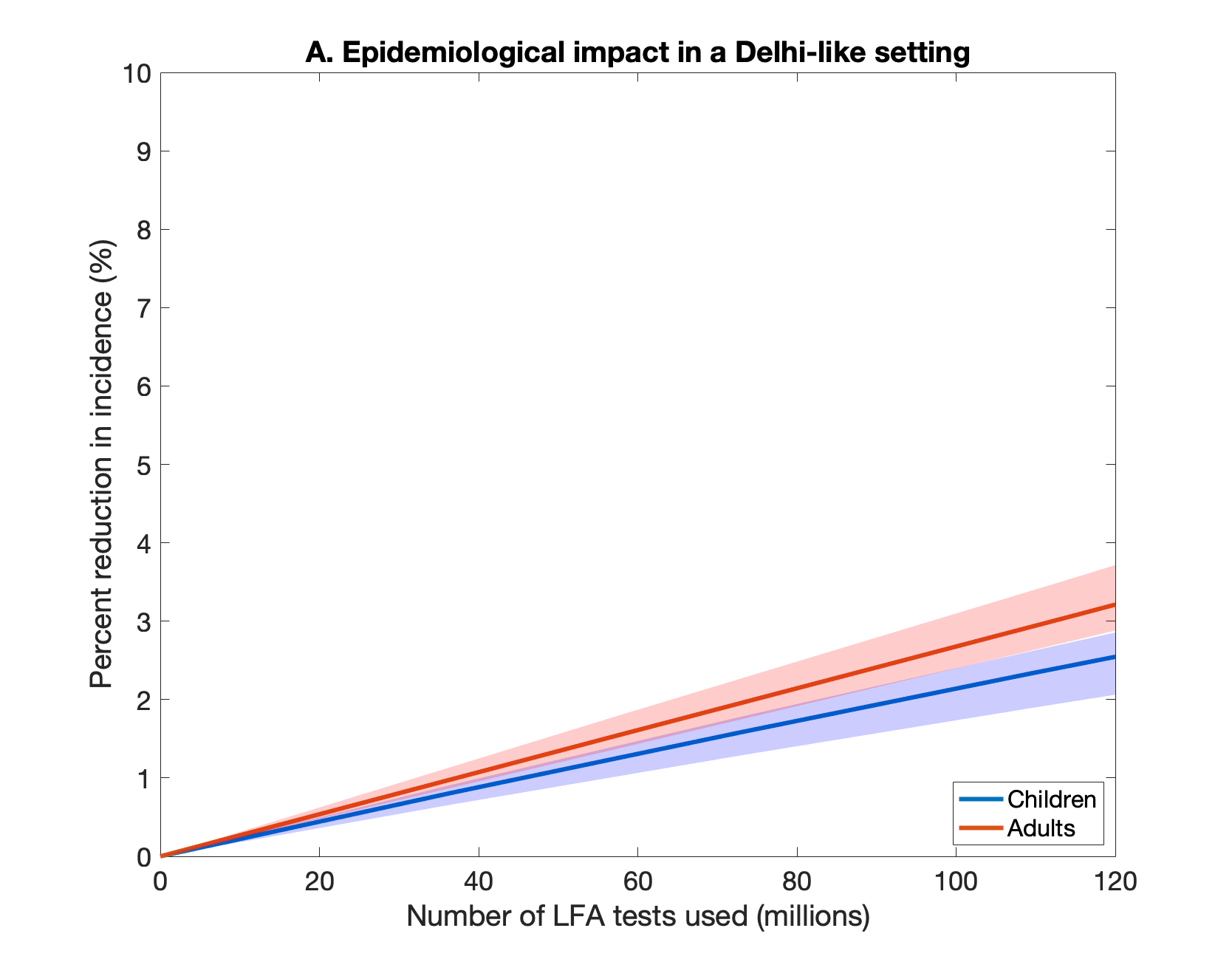

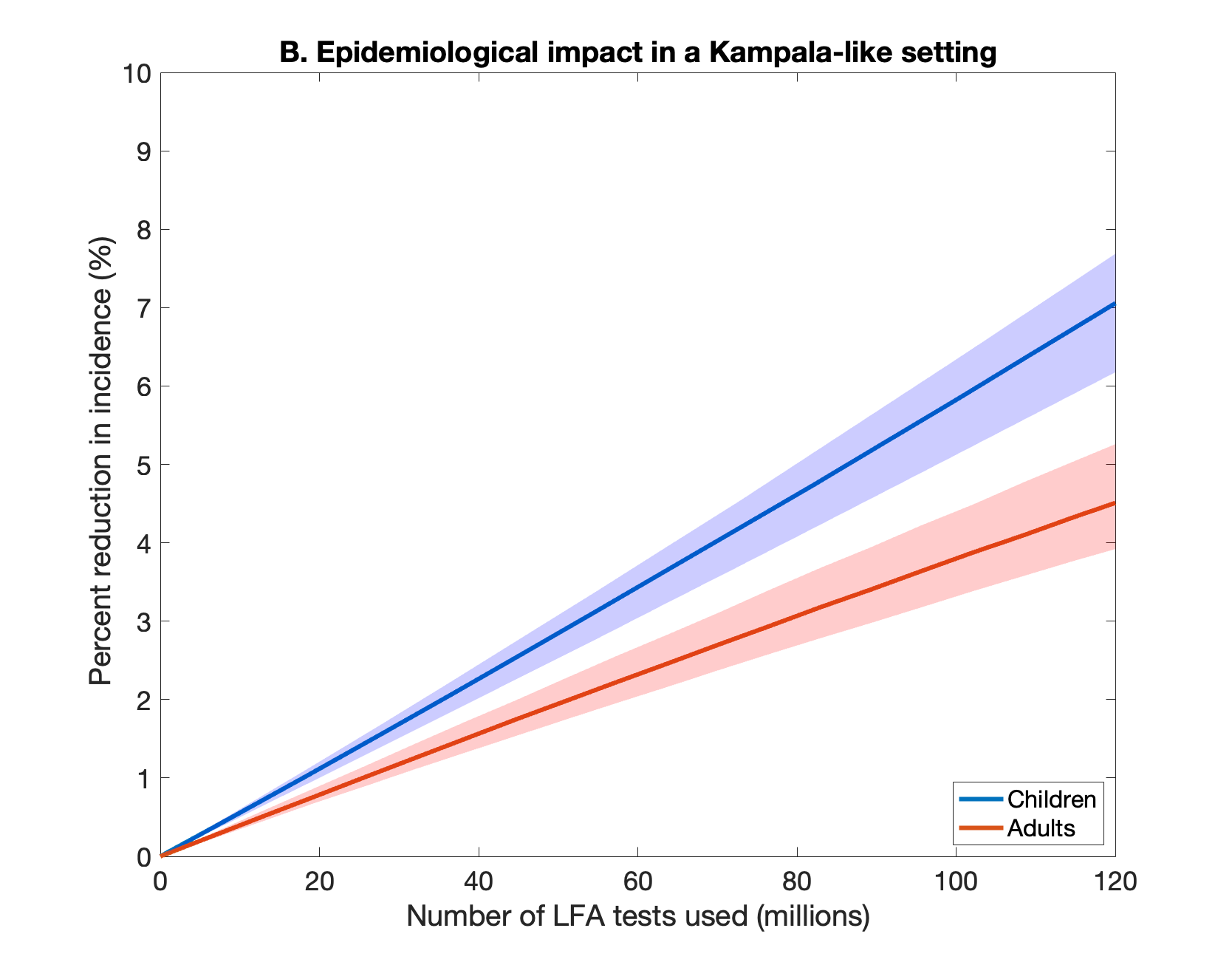


# **4. References**

1. Prem K, Zandvoort K van, Klepac P, et al. Projecting contact matrices in 177 geographical regions: An update and comparison with empirical data for the COVID-19 era. PLOS Comput Biol **2021**; 17:e1009098. Available at: https://doi.org/10.1371/journal.pcbi.1009098.

2. Ritchie H, Mathieu E, Rodés-Guirao L, et al. Coronavirus Pandemic (COVD-19). 2020.

3. He X, Lau EHY, Wu P, et al. Temporal dynamics in viral shedding and transmissibility of COVID-19. Nat Med **2020**; 26:672–675. Available at: https://doi.org/10.1038/s41591-020-0869-5.

4. Prevention C for DC and. Interim Clinical Guidance for Management of Patients with Confirmed Coronavirus Disease (COVID-19). 2021.

5. Mina MJ, Parker R, Larremore DB. Rethinking Covid-19 Test Sensitivity — A Strategy for Containment. N Engl J Med **2020**; 383:e120. Available at: https://doi.org/10.1056/NEJMp2025631.

6. Beigel JH, Tomashek KM, Dodd LE, et al. Remdesivir for the Treatment of Covid-19 — Final Report. N Engl J Med **2020**; 383:1813–1826. Available at: https://doi.org/10.1056/NEJMoa2007764.

7. Davies NG, Klepac P, Liu Y, et al. Age-dependent effects in the transmission and control of COVID-19 epidemics. Nat Med **2020**; 26:1205–1211. Available at: https://doi.org/10.1038/s41591-020-0962-9.

8. Li W, Su Y-Y, Zhi S-S, et al. Virus shedding dynamics in asymptomatic and mildly symptomatic patients infected with SARS-CoV-2. Clin Microbiol Infect **2020**; 26:1556.e1-1556.e6. Available at: https://pubmed.ncbi.nlm.nih.gov/32653662.

9. Lee S, Kim T, Lee E, et al. Clinical Course and Molecular Viral Shedding Among Asymptomatic and Symptomatic Patients With SARS-CoV-2 Infection in a Community Treatment Center in the Republic of Korea. JAMA Intern Med **2020**; 180:1447–1452. Available at: https://doi.org/10.1001/jamainternmed.2020.3862.

10. Margolin E, Burgers WA, Sturrock ED, et al. Prospects for SARS-CoV-2 diagnostics, therapeutics and vaccines in Africa. Nat Rev Microbiol **2020**; 18:690–704. Available at: https://doi.org/10.1038/s41579-020-00441-3.

11. Gupta N, Bhatnagar T, Rade K, Murhekar M, RR G, Nagar A. Strategic planning to augment the testing capacity for COVID-19 in India. PG - 210-215 LID - 10.4103/ijmr.IJMR_1166_20 [doi].

12. Carter LJ, Garner L V, Smoot JW, et al. Assay Techniques and Test Development for COVID-19 Diagnosis. ACS Cent Sci **2020**; 6:591–605. Available at: https://doi.org/10.1021/acscentsci.0c00501.

13. Böger B, Fachi MM, Vilhena RO, Cobre AF, Tonin FS, Pontarolo R. Systematic review with meta-analysis of the accuracy of diagnostic tests for COVID-19. Am J Infect Control **2021**; 49:21–29. Available at: https://pubmed.ncbi.nlm.nih.gov/32659413.

14. EMERGENCY USE AUTHORIZATION (EUA) SUMMARY COVID-19 RT-PCR TEST. 2021. Available at: https://www.fda.gov/media/136151/download.

15. Sethuraman N, Jeremiah SS, Ryo A. Interpreting Diagnostic Tests for SARS-CoV-2. JAMA **2020**; 323:2249–2251. Available at: https://doi.org/10.1001/jama.2020.8259.

16. Antigen-detection in the diagnosis of SARS-CoV-2 infection using rapid immunoassays. 2020. Available at: https://www.who.int/publications/i/item/antigen-detection-in-the-diagnosis-of-sars-cov-2infection-using-rapid-immunoassays.

17. Sharma N, Sharma P, Basu S, et al. The seroprevalence and trends of SARS-CoV-2 in Delhi, India: A repeated population-based seroepidemiological study. medRxiv **2020**; :2020.12.13.20248123. Available at: http://medrxiv.org/content/early/2020/12/14/2020.12.13.20248123.abstract.

18. Uyoga S, Adetifa IMO, Karanja HK, et al. Seroprevalence of anti–SARS-CoV-2 IgG antibodies in Kenyan blood donors. Science (80- ) **2021**; 371:79 LP – 82. Available at: http://science.sciencemag.org/content/371/6524/79.abstract.

19. Population Pyramids of the World from 1950 to 2100, India 2020. 2019.

20. Delhi Population 2020.

21. Population pyramids of the World from 1950 to 2100, Uganda 2020. 2019.

22. Kampala Population 2020.
